# Obstructive Sleep Apnea and Right Ventricular Remodeling: Do We Have All the Answers?

**DOI:** 10.3390/jcm12062421

**Published:** 2023-03-21

**Authors:** Marijana Tadic, Cesare Cuspidi

**Affiliations:** 1 Klinik für Innere Medizin II, Deparment of Cardiology, Universitätsklinikum Ulm, Albert-Einstein Allee 23, 89081 Ulm, Germany; 2 Department of Medicine and Surgery, University of Milano-Bicocca, 20125 Milano, Italy

**Keywords:** obstructive sleep apnea, right ventricle, systolic function, longitudinal strain, continuous positive airway pressure therapy

## Abstract

Obstructive sleep apnea (OSA) syndrome is a very important sleep-related breathing disorder related to increased cardiovascular and overall morbidity and mortality. It is associated with multisystemic target organ damage due to micro- and macrovascular changes, resulting in carotid and coronary atherosclerosis, increased arterial stiffness, retinal damage, microalbuminuria, and cardiac remodeling. The latter consists of left ventricular (LV) hypertrophy, as well as diastolic and systolic dysfunction. The increasing burden of evidence shows that OSA also induces right ventricular (RV) remodeling that is more difficult to diagnose, but may also contribute to cardiovascular morbidity and mortality in these patients. Conventional echocardiographic parameters for assessment of RV systolic and diastolic functions are often not sensitive enough to detect subclinical and subtle changes in the RV function. Data published over last decade showed that the RV function, particularly systolic, is impaired in OSA patients and related with its severity. However, the introduction of speckle tracking echocardiography and the particularly longitudinal strain enabled the earlier detection of functional and mechanical changes even when conventional echocardiographic parameters of RV systolic function remained unchanged. The 3D echocardiography provided the possibility to evaluate the entire RV, with its unique shape, and determine 3D RV ejection fraction, which is comparable with results obtained by cardiac magnetic resonance. The use of this modality also provided a new insight into RV systolic (dys)function in OSA patients. In addition to weight loss, which has been proven very helpful in OSA patients, the only approved therapeutic approach is continuous positive airway pressure (CPAP) therapy. It is very important to assess if this therapy induces any improvement in cardiac structure and function. Limited data on this topic show that RV longitudinal strain is a more sensitive parameter rather than other conventional RV indexes in the detection of improvement in RV systolic function and mechanics. The aim of this review article is to summarize the current understanding of RV structural, functional, and mechanical changes in patients with OSA. Furthermore, we sought to provide the current knowledge regarding the effect of CPAP therapy on RV reverse remodeling in OSA patients.

## 1. Introduction

Obstructive sleep apnea (OSA) syndrome is the most frequent sleep-related breathing disorder that has been the most prevalent in middle age population of patients suffering from obesity, diabetes, and arterial hypertension. It has been related with changes in pulmonary circulation that result in right ventricular (RV) structural and functional changes, which may be related with poor outcomes in these patients [1,2,3]. Overnight polysomnography is used as a standard technique for OSA diagnosis and quantification. Apnea-hypopnea index (AHI) represents the number of apnea and/or hypopnea episodes per hour. OSA patients are classified into mild (5 < AHI < 15), moderate (15 < AHI < 30), and severe (AHI > 30).

Studies showed that patients with OSA experienced higher cardiovascular morbidity and mortality in comparison with the general population [4,5], which is why this population of patients became of great importance for the medical community over the last decade. The more recent studies showed that continuous positive airway pressure (CPAP) therapy significantly reduced cardiovascular morbidity and mortality over the period of 15 years [4].

RV changes are the results of oxidative stress, inflammation, elevated increased sympathetic nervous activity, and consequently elevated blood pressure, both systemic and pulmonary. Furthermore, the high negative intrathoracic pressures have an unfavorable effect on pulmonary and systemic hemodynamics, mainly by increasing afterload [6]. The remodeling is reflected in RV hypertrophy and RV systolic dysfunction, but the latest studies revealed that the RV longitudinal strain represents a better early imaging marker for RV remodeling than other conventional echocardiographic parameters that have been routinely used for the evaluation of RV structure and systolic function. It seems that RV longitudinal strain is more sensitive to subclinical changes in RV function than other tricuspid annular plane systolic excursion (TAPSE), s’ (systolic flow velocity of the lateral tricuspid annulus measured by tissue Doppler), and fractional area change (FAC) [7,8]. This makes it more appropriate for easy and prompt detection of subclinical RV damage, as well as for the monitoring of RV functional improvement during CPAP therapy, which was proven to be very effective in these patients [4,5,9].

The PubMed, OVID-MEDLINE, and Cochrane library databases were analyzed to search for English-language articles published until December 2022. Studies were identified by using the following terms: “obstructive sleep apnea”, “sleep quality”, “sleep disordered breathing”,“cardiac damage”, “right ventricle”, “systolic dysfunction”, “global longitudinal strain”, ”right ventricular mechanics”, “echocardiography”, and “STE echocardiography”.

The aim of this review article is to provide a summary of the current knowledge about RV structural, functional, and mechanical remodeling in patients with OSA, as well as the effect of CPAP on RV reverse changes in these patients.

## 2. Mechanisms of RV Remodeling in OSA

There are several mechanisms that can be responsible for changes in RV morphology, function, and mechanics in OSA patients. Many investigations reported permanently increased pulmonary pressure and even pulmonary hypertension in these patients. This may be related with chronic obstructive pulmonary disease, but also with cardiac comorbidities, such as left ventricular (LV) dysfunction and hypertension. Nevertheless, even in the absence of other known cardiopulmonary disorders, permanent pulmonary hypertension can develop in these patients [10], which can further induce the development of RV hypertrophy and dysfunction. Obese patients with hypoventilation syndrome have a higher risk of obtaining pulmonary hypertension due to the additional negative impact of hypercapnia, with a prevalence 59% greater than that among those with isolated OSA [11].

Early data reported that RV dysfunction in OSA patients was related with daytime hypoxemia due to underlying lung disease [12], but recently published studies showed that the RV function is impaired in OSA patients even after adjustments for all potential confounders [13]. An independent effect of OSA on RV structure and performance was observed in a number of studies, showing a correlation between apnea-hypopnea index (AHI) and RV parameters. A frequently mentioned mechanism of RV remodeling in OSA patients is obesity, which is frequently seen in this population. Namely, OSA is present in about 40% of obese individuals, whereas approximately 70% of OSA patients are obese [14]. The generation of negative intrathoracic pressure against an occluded airway might be the mechanism that could lead to RV dysfunction in OSA patients because it induces increased venous return and RV volume overload during apnea periods [10]. RV hypertrophy is increasing myocardial oxygen demands, which may lead to RV ischemia and dysfunction [15].

### 2.1. RV Structure

RV diameters are increased in OSA patients compared with control subjects in the majority of studies investigating the effect of OSA on RV remodeling [16,17,18,19]. RV thickness has been significantly less evaluated in available studies, but existing results revealed significantly higher RV thickness in patients with OSA than in control group [16]. However, the authors did not find a correlation between OSA severity, measured by AHI, and RV thickness. The prevalence of RV hypertrophy did not differ between OSA and control groups (18.3% vs. 20%) [16]. The same group of authors reported that the presence of OSA was significantly related with RV diameter and thickness, and it was independent of potential confounders [16]. RV dilatation was observed in patients with OSA as a group, but there was no significant difference in RV diameters between patients with various levels OSA severity [16]. However, other authors reported a significant difference in RV diameter between patients with moderate-severe OSA and participants with mild OSA [17]. Nevertheless, there are also authors who did not find any difference in RV diameter or thickness between OSA patients and controls [13,20,21,22,23]. A 3D RV evaluation revealed that RV volumes (end-diastolic and end-systolic) gradually and significantly increased from the patients with mild OSA to those with moderate and severe OSA [21]. A large meta-analysis that included 902 OSA patients and 596 control subjects reported significantly higher RV diameter and RV wall thickness in OSA patients [24]. Table 1 summarizes RV structural, diastolic, and systolic function parameters in OSA patients in available studies.

### 2.2. RV Diastolic Function

The evaluation of RV diastolic function and filling pressures are not frequently assessed in studies investigating OSA patients, and available data are conflicting (Table 1). Peak early diastolic tricuspid annular velocity was similar between participants with moderate and severe OSA and subjects with mild OSA or controls [20]. Some authors reported a gradual increase in tricuspid E/A and E/e’ ratio from patients ranging from mild to moderate to severe OSA [18,25], which was confirmed even with RV diastolic strain rates. Both groups of authors found a significant correlation between OSA severity represented by AHI and parameters of RV diastolic function (E, A, DT, E/A, E/e’) [18,25]. Vitarelli et al. found a significant difference only between severe and mild OSA patients [23]. However, the authors reported a gradual increase in tricuspid E/e’ that did not reach statistical significance mainly due to small sample size (10 mild, 8 moderate, and 19 severe OSA patients). It is difficult to compare different studies in OSA patients, as the authors used various parameters for the evaluation of RV diastolic function. Additionally, it is questionable which parameter of RV diastolic function is more sensitive to changes of RV filling pressures and which should be used at the first line to detect subtle changes in RV diastolic function in these patients. Namely, Güvenç et al. did not find any difference in tricuspid E/A, though they found a significant difference in E/e’ [22]. Many authors did not find a significant difference in pulsed and tissue Doppler parameters of RV diastolic function between OSA and control subjects [13,20,21,26]. The difference was not found even when OSA patients were divided into two groups according to systolic pulmonary pressure (cut-off was 30 mmHg) [26].

### 2.3. RA Remodeling

There is not much evidence about RA changes in OSA patients, and findings are conflicting. Some authors reported dilated RA in OSA patients [19,25,28] and even gradual enlargement in RA volume index from mild, across moderate, to severe OSA [28]. On the other hand, there are authors who did not find a difference in RA size (area or volume) between OSA and control subjects [13,20,22].

The RA phasic function is a more comprehensive method for evaluation of RA remodeling, as it provides information about RA function during each part of the cardiac cycle. It has three phases: reservoir during ventricular systole, conduit during early ventricular diastole, and booster pump during late ventricular diastole [29]. RA reservoir function involves RA relaxation and compliance, while RA conduit function is related with RV diastolic function and RV relaxation. RA contractile function represents intrinsic RA contractility and RV end-diastolic compliance and pressure. There are two methods for evaluation of RA phasic function: volumetric and strain method. The latter is used more frequently nowadays because of its higher sensitivity, better reproducibility, and faster analysis [29].

Li et al. investigated RA phasic function in OSA patients, using velocity vector imaging, and revealed no difference in reservoir function between patients with various level of OSA severity, whereas conduit function was lower and booster pump function was higher in patients with moderate and severe OSA comparing with controls [28]. Early and late diastolic RA rates, illustrating RA conduit and pump function gradually reduced from mild to severe OSA, shows a small discrepancy between different techniques used for the assessment of RA phasic function (volumetric vs. strain evaluation). However, the number of included patients is limited, and these kind of differences are negligible. These results reflect the correlation between the severity of OSA, measured by AHI, and RA phasic function parameters, particularly conduit function [28]. RA global longitudinal strain (GLS) was independent of clinical and echocardiographic characteristics associated with AHI, which confirmed the importance of influence of OSA severity on RA remodeling and RV diastolic function. Similar results regarding the correlation between AHI and RA volume index were reported by the other authors [25].

### 2.4. RV Systolic Function

Different parameters were used for the evaluation of the RV systolic function in OSA patients (Table 1). Conventional parameters mostly include TAPSE and s’, whereas more a comprehensive approach involves evaluation of FAC and myocardial performance index (Tei index). The most advanced investigations used 3D echocardiography for the assessment of 3D RV ejection fraction (RVEF) in the evaluation of RV systolic function [17,23]. The results are not uniform, but the majority of studies agree that RV systolic function is deteriorated in OSA patients comparing with controls, even though the values remain in the normal range [16,17,18,19,23,25,26,30] (Table 1). It should be also mentioned that there was a discrepancy between various parameters, which means that some of them (usually TAPSE) were reduced and others preserved or at least did not reach statistical significance to prove difference between OSA and control participants [19,23,25,26]. Some authors interpretated these results as the consequence of small sample size, while others suggested that one echocardiographic parameter was more sensitive than other, which is difficult to justify in a small sample size.

Vitarelli et al. reported not only the reduction in TAPSE, FAC, and 3D RVEF in patients with moderate and severe OSA, but also a significant difference in 3D RVEF between OSA patients with and without pulmonary hypertension [23]. However, there are also investigators who reported no significant difference in RV systolic function between OSA and control subjects [13,20,21,27].

The meta-analysis that included a large number of OSA patients and evaluated all conventional parameters of RV systolic function (myocardial performance index, s’, TAPSE, and FAC) showed a significant increase in myocardial performance index and decrease in all other parameters, which only confirms the impairment of RV systolic function in OSA patients [24].

The difference was also noticed when correlation between OSA severity, defined by AHI, and RV systolic function was investigated. Some studies reported a significant correlation and worse RV systolic function in patients with moderate and severe OSA [18,25,30], whereas others did not find any significant relationship between severity of disease and RV systolic function [16,21,27]. Vitarelli et al. did not find that reduction in TAPSE or FAC can predict severe OSA (AHI > 30), whereas 3D RVEF was the only parameter of RV systolic function which could predict severity of OSA with sensitivity of 89% and specificity of 77% [23]. The other study using 3D echocardiography showed that the prognostic value of echocardiographic parameters for the evaluation of RV systolic function in prediction of OSA declines from 3D RVEF (AUC 0.94), across TAPSE (AUC 0.92) and FAC (AUC 0.90), to s’ (AUC 0.85), with the lowest predictive value [17].

### 2.5. RV Mechanics

RV global and free-wall longitudinal strains represent parameters of RV systolic function and mechanics, which provide more information than conventional parameters (TAPSE, FAC, s’, and myocardial performance index) due to their higher sensitivity, specificity, reproducibility, and predictive value in a large spectrum of cardiovascular conditions [7,8,9]. Table 2 summarizes data about RV GLS and free-wall RVLS at baseline and strain values, as well as RV systolic parameters after CPAP therapy.

Investigations mostly agree about reduced RV GLS and free-wall LS in OSA patients [13,17,23,26,27,30,31,33], and most of them speculate about higher sensitivity to recognize subclinical RV damage in patients with preserved parameters of RV systolic function (Table 2). Additionally, authors reported significant difference in RV longitudinal strain values between patients with various OSA severity [23,30], which also contributes to the claims that strain is more sensitive to subtle myocardial changes. However, some investigations showed a gradual deterioration between patients with mild, moderate, and severe OSA only in free-wall RVLS, but not in RV GLS, which remained the most impacted only in patients with severe OSA [23]. Buonauro et al. showed the difference in RV GLS and free-wall RVLS, but not in septal LS, which emphasizes the importance of OSA influence on the RV independently of LV [26]. Furthermore, the authors did not find a difference in TAPSE, s’, and 3D RVEF between OSA patients and controls, but only in RVLS, which supports the hypothesis that strain is more sensitive to parameters than other echocardiographic indices used in daily clinical routine [26]. RV GLS and free-wall RVLS were also able to discriminate OSA patients with and without pulmonary hypertension. AHI was independent of age, BMI, and systolic blood pressure associated with both RV GLS and free-wall RVLS.

Some authors went so far as to investigate RV segmental changes in OSA patients, and interestingly found no difference in RV basal and apical free-wall LS, even though average free-wall RVLS was significantly lower in OSA patients [33]. All septal segments showed significantly lower LS in OSA patients, but septum is also part of the left ventricle, and it is difficult to distinguish OSA influence on the left and right ventricle separately. AHI did not correlate with any segmental RVLS in the whole population, but AHI correlated with mid-free-wall RVLS in a subgroup of patients under 60 years old, and with basal septal RVLS only in women [27]. On the other hand, Hammerstingl et al. reported lower RVLS in all three segments of the free wall and found a significant correlation between AHI and RV GLS [27]. The same group of authors reported a significant difference in RVGLS, apical, and basal RVLS between patients with different OSA severity [31]. The most extensive gradual deterioration was detected in apical RVLS. Interestingly, the investigators did not find any difference in TAPSE, s’, or the myocardial performance index between OSA and control subjects or patients with different levels of OSA severity [27,33]. The investigation that used velocity vector imaging revealed a significant reduction in RV longitudinal strain and strain rate of free-wall basal, mid, and apical segments [30]. The negative impact of OSA on RV strain and strain rates was significantly dependent on its severity. The same method used by the other research group confirmed these findings, but only for the basal and mid segments of RV free wall [13]. Tissue-Doppler-derived strain and strain rates performed for all RV segments showed that only apical free-wall RVLS was lower in OSA patients and gradually decreased with OSA severity, whereas all other segments did not show either a significant impairment in OSA subjects or a difference between mild, moderate, and severe OSA [21]. However, velocity vector imaging and tissue-Doppler-derived strain are replaced by a speckle tracking-derived strain, and all other studies were based on this method. Therefore, it is difficult to compare these investigations [21,27,29,31] with the rest of studies on the same topic.

RVLS shows a significant predictive value in the diagnosis of severe OSA (AHI > 30). Vitarelli et al. showed similar sensitivity and specificity of 3D RVEF and RVLS to detect severe OSA [23], whereas Chetan et al. showed not only that 3D RV GLS was significantly lower in OSA patients, but also that this parameter has the highest predictive value in OSA diagnosis including all echocardiographic parameters [17].

It should also be noted that not all investigators documented a reduced RVLS in OSA patients. In a very specific population of OSA patients living at high altitude (1768 m above sea level) there was no difference in free-wall RVLS, TAPSE, s’, or 3D RVEF [22]. The obtained values for these parameters were almost super normal, which may be related with lifestyle and high altitude.

Our meta-analysis that included almost all aforementioned studies showed significantly lower RV GLS and free-wall RVLS in OSA patients compared with controls, as well as in patients with severe OSA in comparison to those with mild OSA [8]. The meta-regression analysis showed a significant association between AHI and RV GLS in all included OSA patients [8]. It should be also noted that TAPSE was reduced in OSA patients included in this meta-analysis, but it was not able to differentiate mild from moderate or severe OSA. This was also the case with RV GLS, but not with the free-wall RVLS that was able to distinguish mild from moderate or severe OSA [8]. These findings are encouraging the evaluation of RV longitudinal strain, particularly free-wall RVLS, in all OSA patients at any stage of disease, as it was proven to be a sensitive parameter of subclinical RV damage (Table 2).

### 2.6. The Effect of CPAP on Reverse RV Remodeling

CPAP represents the most effective method for OSA treatment, usually in combination with dietary changes that are crucial for the reduction in body weight, which sometimes includes extreme procedures such as bariatric surgery. In addition to improv quality of life and some polysomnographic parameters, it is difficult to determine the level of improvement caused by CPAP. Novel echocardiographic parameters, primarily RV strain, can help the clinician in determining reverse cardiac remodeling (Table 2).

Vitarelli et al. showed that CPAP therapy after 4 months induced improvement in 3D RVEF, RV GLS, and free-wall RVLS, but not in TAPSE, FAC, and s’ [23]. The AHI decreased significantly, mean and lowest arterial oxygen saturations increased, and duration of oxygen saturation <90% significantly decreased after CPAP therapy. Another investigation revealed that RV GLS, s’, and FAC significantly improved after 3-month CPAP therapy, whereas free-wall RVLS did not significantly change over the same period [32]. On the other hand, all types of LV longitudinal and circumferential strains in both layers (endocardial and epicardial) significantly improved over the course of CPAP therapy. This would imply that CPAP therapy has significantly faster and more effective influence on the LV than on the RV. Considering the fact that conventional 2D and Doppler methods for evaluation of the LV remained unchanged after 3 months of therapy, these findings suggested that speckle-tracking echocardiography is more sensitive for the detection of subtle changes in LV longitudinal function [32]. An investigation that compared the influence of non-invasive ventilation (NIV) and CPAP in OSA patients showed that RV GLS, as well as free-wall RVLS (average and each segment separately), significantly deteriorated during NIV therapy and improved during 6–8 months of CPAP therapy [20]. TAPSE and s’ did not change in OSA patients treated with any of these two modalities, which illustrates a higher sensitivity of strain and speckle tracking imaging than conventional echocardiographic RV parameters. Interestingly, arterial blood gasses changed to the same levels with NIV and CPAP.

Hammerstingl et al. showed that only apical free-wall RVLS significantly improved after 6 months of CPAP therapy, whereas other segments and RV GLS did not change [31]. When apical free-wall RVLS was analyzed in all OSA patients separated into three groups depending on OSA severity, only patients with moderate and severe OSA showed improvement after CPAP therapy. In all these studies, BMI did not significantly change during the follow-up period, and therefore cannot influence on LV or RV changes.

Our recent meta-analysis showed that CPAP did not significantly change TAPSE, but significantly improved RV GLS [9]. Simultaneously, we showed that this type of therapy significantly improved LVEF and LV GLS. There are many inconsistencies regarding age, BMI, OSA duration, and severity, as well as comorbidities between patients in the included studies, which is why we should consider these findings with caution (Table 2).

## 3. Clinical Impact

OSA is associated with increased cardiovascular and overall morbidity and mortality, which is largely related to cardiac damage. The main issue is to diagnose these subclinical changes as soon as possible, as well as to detect cardiac improvement soon after CPAP therapy is initiated. An increasing body of evidence shows that conventional echocardiographic parameters are not sensitive enough, and new imaging parameters such as speckle tracking derived longitudinal strain have better sensitivity and predictive value in OSA patients. Therefore, RV GLS and particularly free-wall RVLS should be considered as additional parameters in the routine echocardiographic report in all OSA patients at the baseline, as well as during each follow-up visit. Additional analysis is necessary to determine its real clinical value, particularly its relationship with outcome in OSA patients. Naturally, these measurements should be performed along with complete LV echocardiographic assessment, including LV GLS. On the other side, patients with severe OSA often have comorbidities such as heart failure and/or atrial fibrillation, which can significantly impact the evaluation of RV (dys)function. It should be also acknowledged that even though treatment of OSA is recommended, large, randomized data that suggest a mortality benefit in these patients with treatment/CPAP use are still missing. This remains an important task for future investigation.

## 4. Conclusions

RV longitudinal strain, both global and free-wall, represents a good parameter of subtle RV damage that can be detected before impairment of any other conventional echocardiographic index. Existing studies are very heterogeneous with regards to number of patients, age, BMI, OSA duration and comorbidities, and subjects in the control group. These are significant limitations in making a final conclusion about the effect of OSA on RV remodeling or influence of CPAP therapy on RV reversal remodeling. Large longitudinal studies with long follow-up are warrantied to provide missing information and answers on many raised questions regarding cardiac remodeling in OSA patients.

## Figures and Tables

**Table 1:** RV structure, diastolic, and systolic function in OSA patients.

					RV Structure and Volume			RV Diastolic Function		RV Systolic Function				
Reference	n	Age (Years)	BMI (kg/m^2^)	AHI (n/h)	RVT	RVD	3D RVV	E/A	E/e’	TAPSE	FAC	s’	MPI	3D RVEF
Tugcu et al. [13]	27	54 ± 10	31.1 ± 5.1	40 ± 22	→	→	/	→	/	→	/	→	/	/
Amor et al. [16]	139	54.1 ± 11	36.9 ± 7.4	/	↑	↑	/	/	/	↓	/	/	/	/
Chetan et al. [17]	69	59 (51–67)	34 (30.8–40.2)	28.6 (18.3; 42.1)	/	↑	/	/	/	↓	↓	↓	/	↓
Li et al. [18]	69	48.3 ± 6.4	29.5 ± 3.5	12.7 ± 2.0	↑	↑	/	↓	↑	↓	↓	/	/	/
		47.9 ± 7.8	29.9 ± 5.8	24.0 ± 3.6										
		49.6 ± 5.6	34 ± 4.0	40.8 ± 5.0										
Harańczyk et al. [19]	50	60.3 ± 10.3	34.1 ± 6.0	37.4 ± 19.2	/	↑	/	/	/	↓	/	→	/	/
D’Andrea et al. [20]	55	67.8 ± 11.2	33.6 ± 6.6	35.1 ± 15.4	→	→	/	↓	↑	→	/	→	/	/
Kepez et al. [21]	85	48.8 ± 8.2 48.6 ± 9.2	28.4 ± 3.4 31.5 ± 4.9	15 ± 13 46 ± 42	/	→	/	/	/	/	/	/	/	/
Güvenç et al. [22]	41	48 ± 9	32.0 ± 4.6	53.4 ± 18.5	→	→	↑	→	↑	→	/	→	/	→
Vitarelli et al. [23]	37	47.9 ± 10.3 47.6 ± 9.1 48.1 ± 10.2	26.9 ± 5.8 27.4 ± 5.5 28.2 ± 6.3	7.1 ± 1.9 19.8 ± 2.7 58.9 ± 9.1	↑	/	↑	/	↑	↓	↓	↓	↓	↓
Maripov et al. [24]	1503	/	/	/	↑	↑	/	/	/	↓	↓	↓	↑	/
Altekin et al. [25]	58	47.0 ± 6.4 46.8 ± 5 46.7 ± 7.6	28.7 ± 3.4 29.1 ± 2.3 29.8 ± 2.4	10.7 ± 2.6 20.5 ± 2.6 58.1 ± 16.3	/	/	/	↓	↑	↓	/	→	↑	/
Buonauro et al. [26]	59	54.4 ± 11.2	33.2 ± 7.2	42.0 ± 24.3	/	↑	→	/	/	→	/	→	/	→
Hammerstingl et al. [27]	154	61.7 ± 12.4	31.1 ± 5.8	35.9 ± 28.4	/	/	/	/	/	/	/	→	→	/
Tadic et al. [8]	337	/	/	/	/	/	/	/	/	→	/	/	/	/

3D RVV—3D right ventricular volume, 3D RVEF—3D right ventricular ejection fraction, AHI—apnea-hypopnea index, BMI—body mass index, E/A—ration between tricuspid early and late flow velocity measured by pulsed Doppler, E/e’—ratio between early tricuspid velocities measured by pulsed and tissue Doppler, FAC—fractional area change, MPI—right ventricular myocardial performance index, OSA—obstructive sleep apnea, RV—right ventricle, RVD—right ventricular diameter, RVEF—right ventricular ejection fraction, s’—systolic velocity of the lateral segment of tricuspid annulus, TAPSE—tricuspid annular plane systolic excursion. (arrows means increase **↑**, decrease **↓**, no change **→**).

**Table 2:** RV mechanics in OSA patients at the baseline and after CPAP therapy.

					Baseline RV Mechanics		RV Systolic Function and Mechanics after CPAP				
Reference	n	Age	BMI	AHI	RV GLS	Free-Wall RVLS	TAPSE	FAC	s’	RV GLS	Free-Wall RVLS
Tugcu et al. [13]	27	54 ± 10	31.1 ± 5.1	40 ± 22	/	↓	/	/	/	/	/
Amor et al. [16]	139	54.1 ± 11	36.9 ± 7.4	/	/	/	→	/	/	/	/
Chetan et al. [17]	69	59 (51–67)	34 (30.8–40.2)	28.6 (18.3; 42.1)	↓	↓					
D’Andrea et al. [20]	55	67.8 ± 11.2	33.6 ± 6.6	35.1 ± 15.4	↓	↓	→	/	→	↑	↑
Kepez et al. [21]	85	48.8 ± 8.2 48.6 ± 9.2	28.4 ± 3.4 31.5 ± 4.9	15 ± 13 46 ± 42	↓	/	/	/	/	/	/
Tugcu et al. [13]											
Güvenç et al. [22]	41	48 ± 9	32.0 ± 4.6	53.4 ± 18.5	→	/	/	/	/	/	/
Vitarelli et al. [23]	37	/	/	/	↓	↓	→	→	→	↑	↑
Altekin et al. [25]	58	47.0 ± 6.4 46.8 ± 5 46.7 ± 7.6	28.7 ± 3.4 29.1 ± 2.3 29.8 ± 2.4	10.7 ± 2.6 20.5 ± 2.6 58.1 ± 16.3	↓	/	/	/	/	/	/
Buonauro et al. [26]	59	54.4 ± 11.2	33.2 ± 7.2	42.0 ± 24.3	↓	↓	/	/	/	/	/
Hammerstingl et al. [27]	154	61.7 ± 12.4	31.1 ± 5.8	35.9 ± 28.4	↓	↓	/	/	/	/	/
Hammerstingl et al. [31]	82	63.3 ± 11.5	30.7 ± 5.5	31.4 ± 26.8	/	/	→	/	→	↑	↑
Kim et al. [32]	26	49.1 ± 11.4	27.8 ± 2.8	64.2 ± 20.5	/	/	/	↑	↑	↑	→
Tadic et al. [9]	337	/	/	/	/	/	→	/	/	↑	/

AHI—apnea-hypopnea index, BMI—body mass index, CPAP—continuous positive airway pressure, FAC—fractional area change, GLS—global longitudinal strain, MPI—right ventricular myocardial performance index, OSA—obstructive sleep apnea, RV—right ventricle, RVD—right ventricular diameter, RVEF—right ventricular ejection fraction, RVLS—right ventricular longitudinal strain, s’—systolic velocity of the lateral segment of tricuspid annulus, TAPSE—tricuspid annular plane systolic excursion. (arrows means increase **↑**, decrease **↓**, no change **→**).

## Data Availability

Not applicable.
